# The effect of combined action observation therapy and eccentric exercises in the treatment of mid-portion Achilles tendinopathy: study protocol for a feasibility pilot randomised controlled trial

**DOI:** 10.1186/s40814-022-00981-w

**Published:** 2022-02-07

**Authors:** Deirdre Ryan, Ebonie Rio, Grainne O’Donoghue, Cliona O’Sullivan

**Affiliations:** 1 UCD School of Public Health, Physiotherapy and Sports Science, University College Dublin, Dublin, Ireland; 2 School of Allied Health, La Trobe University Melbourne, Melbourne, Australia

**Keywords:** Action observation therapy, Mid-portion Achilles tendinopathy, Neuroplasticity, Strengthening, Rehabilitation

## Abstract

**Background:**

Mid-portion Achilles tendinopathy (AT) is a common overuse injury which can be difficult to successfully rehabilitate. Whilst peripherally directed treatment approaches that strengthen the Achilles tendon complex can be efficacious for some individuals, others will continue to experience long-standing pain and functional deficits. Expanding our rehabilitation approach beyond the tendon mechanical properties to include techniques which target the central neurophysiological changes which can occur in chronic injuries, including mid-portion AT, may improve our rehabilitation outcomes. Action observation therapy (AOT) is one such technique which targets central changes and can enhance motor learning. To our knowledge, there is currently no available information on the combined effect of AOT and eccentric exercises in the rehabilitation of mid-portion AT, nor understanding of the feasibility of conducting randomised controlled trials that investigate this combined centrally and peripherally directed treatment approach. This protocol outlines the design of a remotely conducted parallel-group randomised controlled trial comparing the efficacy of combined AOT and eccentric loading exercises versus eccentric loading exercises alone for mid-portion AT.

**Methods:**

Participants recruited throughout Ireland with mid-portion AT will be randomly assigned to one of the following groups: (i) The AOT group will observe videos of the eccentric exercises prior to the physical performance of the eccentric exercises. (ii) The control group will observe videos of landscapes prior to the performance of the eccentric exercises. This is a 12-week daily intervention as per the Alfredson loading protocol and outcome measures will be assessed at baseline, week 6 and week 12. Primary feasibility outcomes will include data on numbers of eligible participants, recruitment and retention rates, along with exercise compliance and acceptability of treatment. The primary clinical outcome measure will be the Victorian Institution Symptom Assessment-Achilles Questionnaire (VISA-A) assessing disability. Secondary clinical outcomes will address the remaining core domains as outlined by the International Scientific Tendinopathy Symposium consensus (ICON group) including pain, participation, functional, physical function capacity, quality of life and psychological factors. Widespread bodily pain and centralised pain features and patient satisfaction levels will also be evaluated.

**Discussion:**

This study will provide scientific direction for future randomised controlled trials exploring the effect of AOT and eccentric exercises in the treatment of mid-portion AT on pain, centralised pain features, motor and non-motor functions, quality of life and patient satisfaction levels. The feasibility of the conducting a study remotely from participant screening to final follow-up assessment will also be provided.

**Trial registration:**

ISRCTN58161116

## Background

Mid-portion Achilles tendinopathy (AT) is a common overuse injury, affecting the tendon 2-7cm proximal to the insertion [1]. The incidence of mid-portion AT is increasing, with an estimation of 2.16 per 1000 patient years and estimated cumulative lifetime prevalence of 6% in the general population [2, 3]. Clinical features of mid-portion AT include activity-related pain, decreased functional capacity and local tenderness on palpation [1]. Tendinopathy is often resistant to treatment and is associated with a high rate of recurrence [4]. A proportion of individuals will continue to have long-standing symptoms, with functional activities and participation abilities being impacted over a course of years [5].

Research has shown exercise loading protocols, specifically eccentric exercises, to be effective in successfully rehabilitating some individuals [5–9] Yet, up to 44% can fail to respond to this form of intervention [10, 11]. Injury to the body or nervous system can lead to neuroplastic changes in both the affected and unaffected regions of the body along with the spinal pathways, circuitry and central nervous system [12–14]. Whilst tendon mechanical properties and muscle strength are targeted by exercises, they do not address the aforementioned neurophysiological changes that can occur during injury [15]. Successful human movement requires an integration of all the components within the hierarchical control system, including the central nervous system, muscles and tendons which form a sensory-motor feedback loop. Alterations within this loop, thus affecting neuromuscular control have been demonstrated in persons with AT [16, 17]. Motor and sensory deficits have been further reported on the contralateral side of injury in participants with tendinopathy, further suggesting involvement of the central nervous system in tendinopathy [18]. Therefore, it is reasonable to consider combining intervention techniques that target both the local musculotendinous changes and central neurophysiological changes to try and achieve greater clinical results.

Neurophysiological findings in recent times have led to the emergence of novel treatment strategies that do address cortical reorganisation, for example action observation therapy (AOT) which involves the systematic observation of rehabilitation movements. This causes a neurophysiological activation of the areas in the brain related to both the planning and execution of movements [19]. These specific neurones are called the Mirror Neurone System and activate through both the observation or physical performance of movements [20]. The excitability of the primary motor cortex is increased during AOT, and movement and motor learning abilities can be facilitated as a consequence [21]. AOT is typically followed by the physical performance of the same movements [22].

AOT has become a well-substantiated therapeutic treatment in the field of neurorehabilitation demonstrating improved functional abilities, walking performance and balance across populations with Cerebral Palsy, Multiple Sclerosis, Stroke, Multiple Sclerosis and Parkinson’s Disease [23–29]. To date, AOT has been much less explored in musculoskeletal patients, despite improvements in motor control, functional abilities, range of movement and pain scores being reported in studies investigating amputees and orthopaedic populations, along with patients with chronic lumbar spine pain [19, 30–33]. As AOT has the potential to offer an additional neuroplastic effect, the authors believe the investigation of this treatment technique is warranted in mid-portion AT.

The overall aim of the study is to evaluate the feasibility of a future larger scale randomised controlled trial in examining the effectiveness of AOT combined with an Achilles tendon eccentric loading protocol in participants with mid-Portion AT. Feasibility and pilot studies are invaluable in the process of development to implementation of complex interventions as identified by the Medical Research Council (MRC) [34].

### Primary objectives:

- To determine the participant recruitment and retention rate
- Ascertain the percentage of potential participants that both meet the eligibility criteria and enrol in the study
- To establish compliance with rehabilitation
- Piloting the methodological procedures, including remote implementation
- Reflect on the AOT intervention protocol and amend if indicated
- To assess the level of acceptability using patient satisfaction and perceived effectiveness of treatment scales. A further qualitative analysis will be undertaken after the study is completed, exploring the participants experience of participating in a telehealth intervention trial, assessing trial procedures, interventions and outcome measures

### Secondary objectives:

- Explore trends in treatment effects, comparing mean pre-post differences in clinical outcome measures across groups to minimum clinically important differences where possible
- Explore the relationship with fear of movement, central sensitisation and AOT
- Conduct a power calculation to determine the numbers needed for a future large-scale randomised controlled trial

## Methods design

**Fig. 1:**
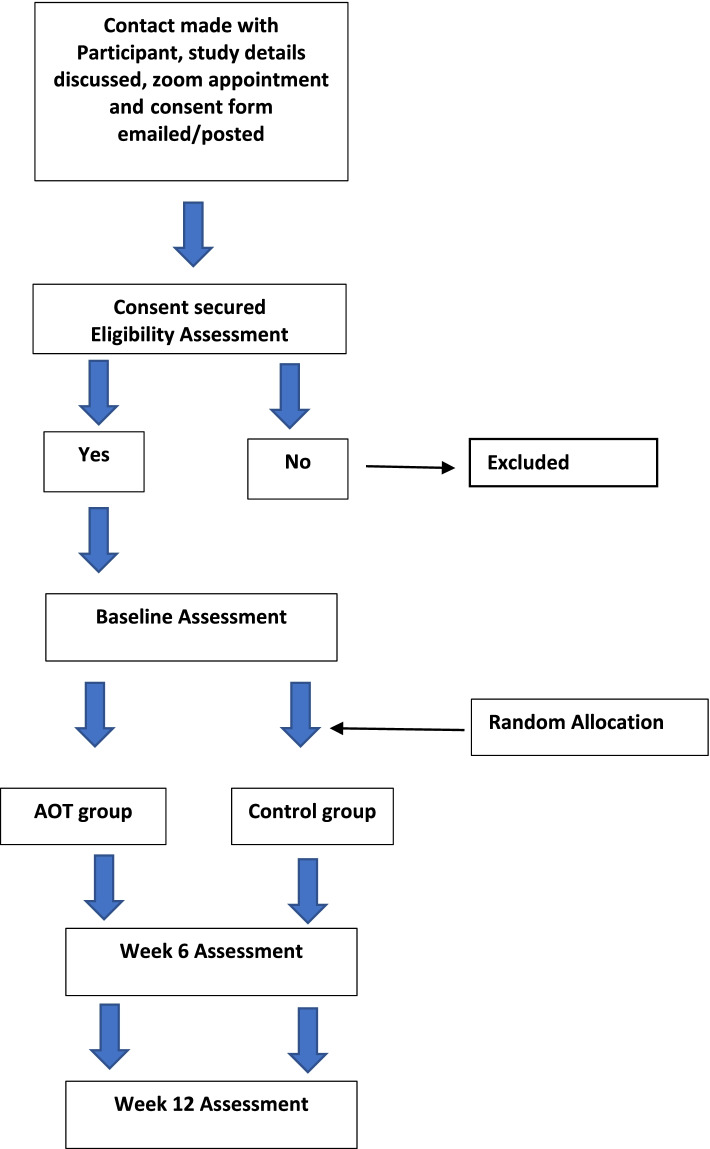
Standard protocol items: recommendation for interventional trials (SPIRIT) flow diagram

This study will adhere to the Standard Protocol Items: Recommendations for Interventional Trial (Fig. 1) [35]. In addition, a qualitative synthesis will explore participants’ perceptions and experiences of the effects of combined AOT and eccentric exercises on the rehabilitation of mid-portion AT.

### Design

This will be a two-group parallel, blinded randomised controlled trial. Both the evaluating therapist and participants will be blinded to group allocations throughout the trial. The study has two arms, the intervention group and control group.

### Setting

This study will be conducted remotely at all stages, with baseline, mid-trial and end of trial assessments to be conducted via zoom.

### Participant recruitment

Participants will be recruited via:

1. Social media platforms including Facebook and Twitter
2. Posters containing study information in sports club websites, gyms, and community centres.
3. Posters containing study information on University College Dublin campus.
4. Emails and letters will be sent to physiotherapy private clinics informing them of the study, with the request to consider referring potentially eligible participants.

### Participant eligibility criteria

Participants will be eligible to participate in the trial if they meet the following criteria:

- Adult aged between 18 and 65, male or female
- Unilateral pain in the mid-portion (2–7cm proximal to insertion) of the Achilles tendon.
- Pain ≥ 3 months.
- Experience morning pain or stiffness.
- Physically active and sedentary populations are included.
- Have access to smart phone, computer, laptop or tablet.
- Competent in written and spoken English and be able to provide consent.

Participants will be excluded from the study should they have any of the following:

- Clinical suspicion of Achilles tendon rupture.
- Previous Achilles tendon surgery in symptomatic leg.
- Bilateral or insertional Achilles tendinopathy.
- Co-existing foot or ankle pathology (os triognum syndrome, retrocalanceal bursitis, superficial calcaneal bursitis, Haglund’s syndrome).
- Systemic disease (e.g. ankylosing spondylitis or rheumatoid arthritis).
- Confounding lower limb injury.
- Metabolic or endocrine disorders, such as type I or II diabetes.
- Corticosteroid injection in/near the Achilles in the last 3 months.
- Condition that prevents the patients from executing an active exercise programme.
- Participant has already performed strength exercise rehabilitation for Achilles pain.
- Use of fluoroquinoline antibiotics within the previous 2 years.

### Screening

**Fig. 2:**
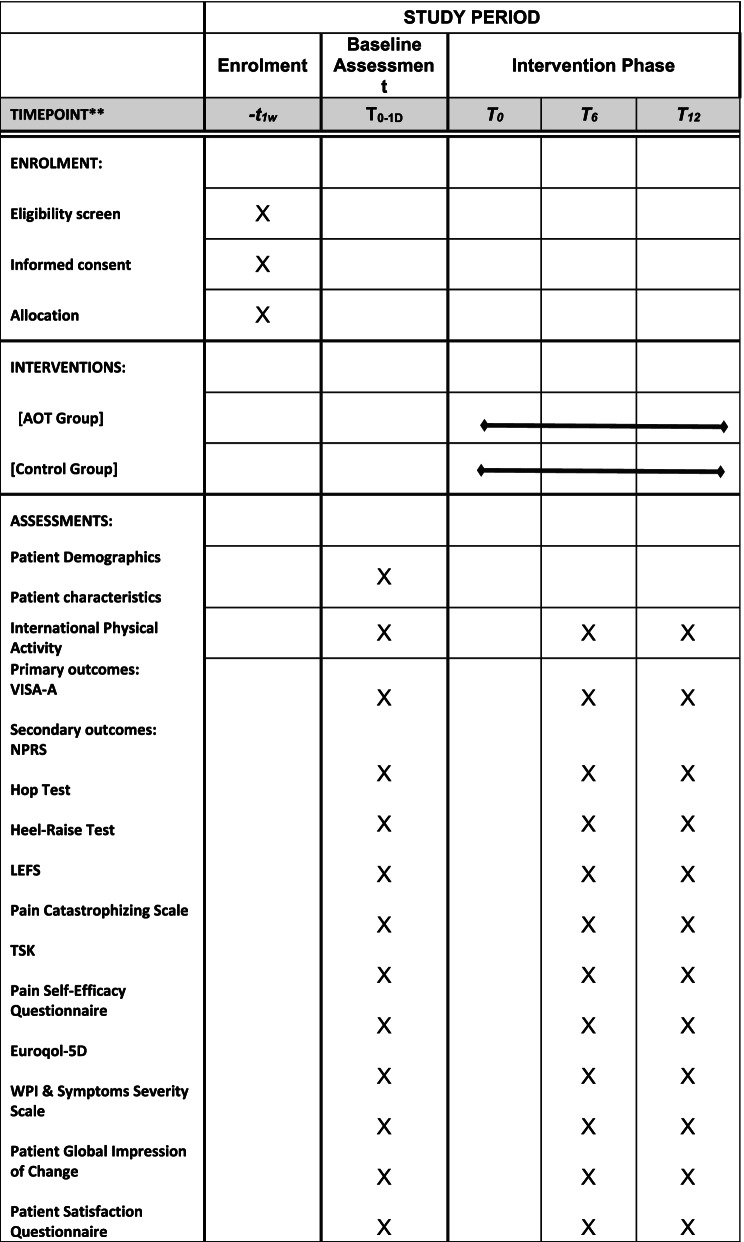
Participant timeline

Initial contact will be over the telephone to organise a time and date for the screening appointment. Participant screening for eligibility will be conducted via zoom; specific questions surrounding the location of Achilles pain using a pain map, the nature of the pain, morning stiffness and pain on the hop test will be assessed. See Participation Timeline (Fig. 2). A link for zoom along with an electronic consent form will be emailed to participant (or posted if preferred)prior to screening. A qualified physiotherapist with over a decade of experience will perform the screening assessment and enrol eligible patients into the study.

### Baseline assessment

Post-screening, a baseline assessment will be conducted. The following will be included in the assessment over zoom:

1. Patient demographics
2. Participant characteristics as outlined by the ICON group [36].
3. Participant exercise habits in a typical week: The International Physical Activity Questionnaire-Short Form provides an estimation of the time spent being physical active and sedentary during the last 7 days. The seven questions measure the time spent sitting, walking, doing moderate and vigorous activities [37].
4. Clinical outcome measures

### Primary clinical outcome measure

#### Disability

- The primary clinical outcome measure will be the Victorian Institution Symptom Assessment-Achilles Questionnaire (VISA-A); consists of 8 questions assessing pain, function and ability to participate is activity. Scores range from 0 to 100, with the higher scores representing a lower clinical severity [38]. The VISA-A has been validated in persons with AT and has robust psychometric properties with excellent reliability reported. As both athletic and non-athletic participants will be recruited, the eight question will use the word ‘physical activity’ to ensure inclusivity. This adjustment has been used previously in research [39].

### Secondary clinical outcome measures

#### Pain over a specified time

- The Numerical Pain Rating Score (NPRS); will be used to quantify worst pain intensity over the past week. The NPRS is rated from 0 to 10, where 0 represents no pain and 10 represents worst pain imaginable, and is preferenced for its superior responsiveness as compared to other pain scales. Accordingly, this scale is considered one of the superior single-item methods available [40].

#### Pain on loading

- Hop test; In standing participants will be instructed to hop with arms relaxed at their side at a self-selected pace on each leg. Participants are aiming for 25 hops and will be asked to rate their pain on the NPRS. Good reliability is associated with this test, with good test-retest reliability (ICC=0.83–0.94) [41].

#### Physical function capacity

- Heel raise for endurance; In standing, participant places 2 fingertips per hand for support against a wall. A maximum number of single-leg heel raises on each side will be assessed. Participants will be instructed to go as high as possible for each repetition and to perform as many repetitions as possible keeping knee straight, trunk upright [42]. Tests will cease when form (achieving at least 50% 1st rep) is unable to be maintained or the movement becomes too painful (greater than 4/10). This test has been shown to have good reliability (ICC=0.76–0.86) [43].

#### Participation

- Lower Extremity Functional Scale (LEFS); a questionnaire containing 20 questions surrounding an individual’s ability to perform everyday tasks and is recommended to assess activity and participation in patients with a diagnosis of mid-portion AT y by current clinical practice guidelines [44]. The LEFS has excellent test-retest reliability (ICC .85–.99), excellent responsiveness and high correlations with similar scales (Pearson correlation value > .07) [45].

#### Participant rating overall condition

- Patient Global Impression of Change; a 7-point scale which assesses a patient’s belief about the efficacy of treatment. The 7-point scale ranges from very much worse to very much improved. This scale possesses high levels of reliability (ICC, *r*=.90) and face validity with values ranging from .72 to .9 [46].

#### Psychological factors

- Pain catastrophising scale; catastrophising can contribute to heightened levels of pain. This 13-item multidimensional scale assesses an individuals’ experience of magnification, rumination and helplessness scale shown to a be a valid andreliable [47].
- Tampa scale for kinesiophobia (TSK); designed for assessment of kinesiophobia, which refers to a fear of movement. The TSK questionnaire comprises of 17 items assessing the subjective rating of kinesiophobia. The score ranges from 17 to 68, with higher scores associated with a  greater fear of movement. This scale has been shown to be a valid and reliable measure in chronic musculoskeletal populations (ICC, *r*=.91) [48].
- Pain self-efficacy questionnaire; a 10-item questionnaire which evaluates an individual’s level of confidence whilst completing certain activities. Scores range from 0 to 60, with higher scores reflective of stronger self-efficacy beliefs. This questionnaire has demonstrated high test-retest reliability (ICC .79–92) and excellent internal reliability (Cronbachs, *α*=.94) [49].

#### Quality of life

- Euroqol-5D; a quality of life questionnaire which examines 5 health-related dimensions including health, mobility, ability to self-care, ability to undertake usual activities, anxiety and depression. Each dimension has five levels, ranging from no problems to extreme problems. This questionnaire has been shown to be both reliable and valid [50].

#### Central pain processing

- The Widespread Pain Index (WPI) and symptoms severity; these measures are part of the fibromyalgia diagnostic criteria with 85% sensitivity and 90% specificity. As central sensitisation is a clinical feature in fibromyalgia patients [51], this tool can be adopted to capture features of central sensitisation in other cohorts of patients. The WPI quantifies the extent of bodily pain on a 0–19 scale, assessing whether patients have pain in 19 different body regions, each point scores 1. The symptom severity scale assesses fatigue, cognitive dysfunction and unrefreshed sleep on a 0–3 scale whereby 0 represents no problem and 3 equates to a severe problem.

### Satisfaction

- Patient satisfaction questionnaire; evaluation of a patient’s satisfaction provides specific feedback which can be utilised by to improve upon the quality and outcomes of patient care [52].

#### Randomisation and allocation concealment

A member of the Research team not involved in the intervention or outcome measure assessments (Researcher 3) will randomly allocate participants to the intervention or control group. The randomisation list will be generated online using a web-based randomisation tool. The number will be placed in an opaque envelope which is given to Researcher 2 whom assigns the intervention or control programmes to each participant.

#### Blinding

All screening and outcome assessments will be performed by a blinded assessor (Researcher 1) online using zoom technology. Participants will be blinded as to their group allocation. Participants will be strongly encouraged not to disclose details regarding their rehabilitation programme during the evaluation assessments at week 6 and week 12.

#### Exercise intervention

**Fig. 3:**
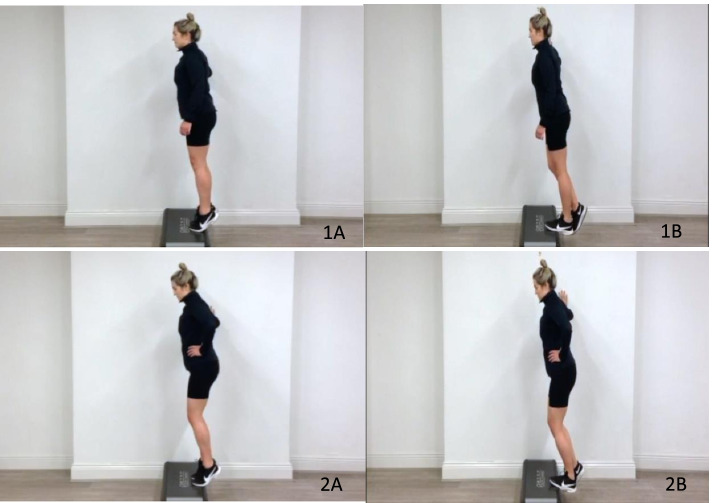
Eccentric loading exercises from the Alfredson Protocol for left mid-portion AT; Exercise 1 (1A+1B) and Exercise 2(2A&2B), both exercises to be performed for 3 sets of 15 repetitions twice a day

Both the AOT and control groups will perform the same physical exercises. The Alfredson eccentric heel drop protocol [6] will be used for the exercise training component (Fig. 3). Post baseline measurements during the initial online assessment, participants will be briefed as to how to correctly perform the two exercises to ensure correct technique throughout the clinical trial. This programme consists of two Achilles eccentric loading exercises to be performed twice a day, for 3 sets of 15 repetitions. Both exercises require the participant to stand on the edge of a step with body-weight on the forefoot. The participant rises on the non-injured leg into plantar flexion and lowers eccentrically on the injured leg, so that the heel lowers below the level of the step. The un-injured leg is then used again to return to the starting position of plantarflexion. The hands are placed on the wall or a railing for balance support. The first exercise is performed with the knee straight, whilst the second exercise is performed with the knee bent (Fig. 3).

#### Video intervention

The AOT and control groups will be provided with different video content, which is to be observed prior to the performance of the exercises. Salaso, an online application used by healthcare providers, will be used for this study. This application allows programmes to be created for patients using videos of exercises, accompanying performance instructions and pdf instructional files (www.salaso.com). Participants will be requested to use the compliance log function in Salaso to record completed rehabilitation sessions. Salaso does not allow exercises to be logged for a given day once the day has past, so the training diary will also be used as a secondary log should participants forget to log through Salaso. This will be viewable by Researcher 2. To facilitate blinding of participants, participants will be informed that they will have to observe videos prior to performing physical exercises.

#### AOT group

Participants in the AOT group will have access to two videos, one of each exercise to be performed (Table 1). The videos will demonstrate a model performing 15 reps of the exercise, the model will match the participants for gender and side of injury. The videos will be observed prior to the physical performance of each set of the exercises. Video 1 is to be viewed prior to performing each set of exercise 1 and video 2 is to be viewed prior to performing each set of exercise 2. Accordingly, each video will be viewed three times, twice a day, daily for 12 weeks.

**Table 1:** Outline of the AOT (Intervention) and Control Group Rehabilitation Programmes

	AOT group	Control group
**Videos**	Two videos; one for each of the eccentric exercises.	Two videos; landscape video 1 and landscape video 2.
**Exercise**	Alfredson eccentric heel drop protocol (2 exercises)	Alfredson eccentric heel drop protocol (2 exercises)
**Method**	Watch video of model perform the eccentric exercise prior to the performance of each set of exercises.	Watch video of landscape prior to the performance of each set of exercises.

#### Control group

The control group will have access to two dynamic landscape videos (with no human or animal content) (Table 1). The two landscape videos will be the same length as the exercise videos. This group will also receive a pdf file in their Salaso account with pictures and instructions as a reminder on how to correctly perform the exercises, the model in the picture will match the participant for gender and side of injury. Landscape video 1 is to be viewed, prior to preforming each set of exercise 1. Landscape video 2 is to be viewed prior to performing each set of exercise 2. Accordingly, each video will be viewed three times, twice a day, daily for 12 weeks.

#### Education and advice

Participants will be requested to not participate in any other form of active treatment during the duration of the study. Each participant will receive standard information regarding mid-portion AT and the exercise rehabilitation programme prior to commencing the intervention along with a training diary calendar. This will be either emailed or posted as per the preference of the participant. The likelihood of Achilles tendon pain or discomfort during the exercises will be explained. With regard to exercise progression, when the eccentric exercises can be performed without any discomfort or pain, participants will be advised to add extra weight. This can be done by wearing a backpack and adding weight to the bag. Participants will be encouraged to do this in 5kg increments. All participants will be requested to document and date the weights added throughout the 12-week trial. Encouragement for continued compliance with the assigned programme will be provided by a monthly email. Participants will be informed to contact the lead Researcher should any problems arise in relation to the exercises.

### Qualitative study

A subset of the participants will be invited to participant in a qualitative study after completing the pilot study. The participants experience of the study will be evaluated, along with levels of satisfaction and barriers and motivators to participation in the study.

### Sample size

As this is a pilot feasibility study, a formal sample size calculation will not be performed. We will aim to have 12 participants minimum per group as a rule of thumb as recommended in the literature [53].

### Statistical analysis

To assess the primary feasibility outcome measures, data pertaining to participant recruitment, withdrawal and completion of intervention will be analysed as percentages. Compliance will also be calculated based upon percentage completion of rehabilitation sessions as collated in Salaso or in the training diaries. An intention to treat analysis will be performed on the clinical outcome measures. Using SPSS software version 24 means and standard deviations (or frequencies and proportions for categorical data), mean or median differences and 95% confidence intervals for clinical outcome measures will be calculated. Although this pilot feasibility study is not powered to detect an effect of treatment, analysis of co-variance (ANCOVA) adjusting for baseline values will be used on the primary clinical outcome measure.

### Progression criteria

The following criteria must be met in order to consider progression to a main RCT:

- A retention rate of ≥ 80% of recruited participants
- A minimum of ≥ 80% of eligible participants enrol in the study

## Discussion

The aim of this study is to evaluate the feasibility of a future larger scale randomised controlled trial examining the effectiveness of AOT combined with Achilles tendon eccentric exercises in the rehabilitation of mid-portion AT. The results from this pilot feasibility study will provide critical data to guide and inform whether a larger-scale randomised controlled trial is warranted. This protocol represents an integrated treatment approach specifically designed to address the possible central and peripheral changes that can occur during mid-portion AT. Whilst potentially providing the needed solution to current rehabilitation programmes to successfully rehabilitate mid-portion AT, this protocol is representative of the MRC guidance for developing and evaluating complex interventions [34]. It is designed to be delivered remotely by physiotherapists and thus allows access to care for patients whom otherwise may not be able to receive such care due to time or transport limitations, along with the national social limitations implemented due to the pandemic.

### Trial status

Recruitment is scheduled to begin in January 2021.

## Abbreviations

AT: Achilles Tendinopathy
AOT: Action observation therapy
VISA-A: Victorian Institution Symptom Assessment-Achilles Questionnaire
ICON: International Scientific Tendinopathy Symposium consensus
NPRS: Numeric Pain Rating Score
LEFS: Lower Extremity Functional Scale
TSK: Tampa Scale for Kinesiophobia
WPI: Widespread Pain Index
